# Prophylactic treatment of migraine; the patient's view, a qualitative study

**DOI:** 10.1186/1471-2296-13-13

**Published:** 2012-03-09

**Authors:** Frans Dekker, Arie Knuistingh Neven, Boukje Andriesse, David Kernick, Ria Reis, Michel D Ferrari, Willem JJ Assendelft

**Affiliations:** 1 Leiden University Medical Center, Public Health and Primary Care, Postzone VO-P, P.O. Box 9600, 2300, RC Leiden, the Netherlands; 2 Headache Lead, Royal College of General Practitioners, London, UK; 3 Amsterdam Institute for Social Science Research University of Amsterdam, Amsterdam, the Netherlands; 4 Neurology LUMC, Leiden, the Netherlands

**Keywords:** Migraine, Prophylaxis, Focus groups, Primary care, Physician-patient relationship

## Abstract

**Background:**

Prophylactic treatment is an important but under-utilised option for the management of migraine. Patients and physicians appear to have reservations about initiating this treatment option. This paper explores the opinions, motives and expectations of patients regarding prophylactic migraine therapy.

**Methods:**

A qualitative focus group study in general practice in the Netherlands with twenty patients recruited from urban and rural general practices. Three focus group meetings were held with 6-7 migraine patients per group (2 female and 1 male group). All participants were migraine patients according to the IHS (International Headache Society); 9 had experience with prophylactic medication. The focus group meetings were analysed using a general thematic analysis.

**Results:**

For patients several distinguished factors count when making a decision on prophylactic treatment. The decision of a patient on prophylactic medication is depending on experience and perspectives, grouped into five categories, namely the context of being active or passive in taking the initiative to start prophylaxis; assessing the advantages and disadvantages of prophylaxis; satisfaction with current migraine treatment; the relationship with the physician and the feeling to be heard; and previous steps taken to prevent migraine.

**Conclusion:**

In addition to the functional impact of migraine, the decision to start prophylaxis is based on a complex of considerations from the patient's perspective (e.g. perceived burden of migraine, expected benefits or disadvantages, interaction with relatives, colleagues and physician). Therefore, when advising migraine patients about prophylaxis, their opinions should be taken into account. Patients need to be open to advice and information and intervention have to be offered at an appropriate moment in the course of migraine.

## Background

Primary care is an important setting for the management of migraine and in many countries most migraine consultations occur in this context [1]. In the Netherlands, migraine is mainly managed in primary care and 95% of prescriptions for triptans are issued in this setting [2].

Prophylactic therapy is an option for patients with frequent or long-lasting migraine headaches [3-8]. The results of 6-12 months of preventive treatment are that in about 50% of patients the attack frequency decreases by 50%. Also, the attacks are often less severe [9]. Drop-out by adverse events is around 5%[10], drop-out due to ineffectiveness is unknown in usual care.

Dutch GP guidelines on headache recommend discussing prophylactic therapy with patients who suffer (on average) 2 or more attacks each month [11]. Despite it being a safe and more or less effective treatment option, only 7-13% of the migraine patients receive it [7,12] and the benefits are not widely accepted. Little is known about the opinions of GPs and patients regarding prophylaxis, or the determinants behind decisions whether or not to start prophylaxis.

This qualitative study explores the opinions, motives and expectations of migraine patients about prophylactic migraine therapy. A similar study focusing on GPs' opinions is reported separately.

## Methods

### Recruitment

Three focus groups of migraine patients were formed, 2 from urban areas and 1 from a rural area. Patient selection was based on pre-specified criteria, aiming to reflect a broad range of experience (from young to old), gender (separate groups for males and females), attack frequency ≥ 2 attacks/months) and pain level (≥ 6 on a scale of 10 matching migraine,1 being almost no headache and 10 being the worst headache ever). Our goal was to achieve a diversity of migraine patients, corresponding to general practice and with a sufficiently high frequency to be eligible for preventive therapy [12,13]. The study was approved by the Ethics Committee of the Leiden University Medical Centre.

One group of 7 females and a second group of 6 males were recruited from 5 urban primary care health centres or group practices. See patient characteristics in Table 1. We selected patients based on the diagnosis migraine and all these patients used prescribed medication for acute treatment. Thirteen patients had consulted their GP or a neurologist for their migraine (2 groups). A third group, comprising 7 females from a rural area, was recruited by a researcher investigating consumer behaviour. In this group each participant was approached by telephone and selected if they had migraine according to the IHS criteria. In this group 2 participants had not received any medical supervision yet.

**Table 1:** Patient characteristics, 2 female and 1 male groups

		Total N = 20	Female 1 n = 7	Male n = 6	Female 2 n = 7
Pain score* (1-10)		8.4	8.2	8.4	8.6
Attack freq./month	2 - 5	16	6	5	5
	≥ 5	4	1	1	2
Age	< 25	4	2	-	2
	25 - 50	11	3	4	4
	> 50	5	2	2	1
	mean	43 yrs	42 yrs	47 yrs	39 yrs
Education level	low	3	-	1	2
	medium	14	5	5	4
	high	3	2	-	1
Paid work (hrs/wk)	none	5	2	1	2
	< 36	8	4	1	3
	≥ 36	7	1	4	2
	mean (hours/week)	22	15	29	21

* Pain level during maximum of attack on a 1-10 numeric scale (10 being unbearable pain)

The application form contained two questions on migraine (severity and frequency), one on the level of education and one about the number of hours in paid work. Based on this application form, the researcher made a comparison with national data on migraine patients in general practice [12]. Regarding the severity and frequency of the migraine, the composition of the three groups corresponded well with the average characteristics of migraine patients in Dutch general practice. The subject mentioned on the invitation was migraine headache in general, without a specific indication of our interest in preventive treatment.

### Data generation

The focus group meetings were chaired by an independent moderator experienced in focus group research. The principal investigator (FD) observed all meetings from an adjacent room via a monitor with sound, but had no influence on the discussions. The moderator used a specially prepared interview guide (compiled by AKN and FD) which started with an introduction and familiarization, followed by discussion on the characteristics of the patients' migraine experience (e.g. age at onset, changes in migraine over time, treatment for attacks, and treatment goals, etc.). Prophylaxis was discussed, including the advantages and disadvantages, and the patients' experiences and attitudes towards preventive medication. In all focus groups sessions a topic list was used, which included some provocative statements to stimulate discussion and the exchange of ideas. The quantitative data listed in the results section are based on this topic list. All sessions were digitally recorded on DVD.

### Data analysis

The recordings were analysed independently by three researchers (FD, AKN and BA). Because the DVD recordings provided the most detailed information on both verbal and non-verbal communication, these served as the primary data source [14-16]. The researchers used regular DVD-reading software with good on-screen forward/backward and other search possibilities. The DVDs allowed both hearing and seeing of non-verbal indications as to whether or not an opinion was supported by others in the group. The three investigators individually identified 'themes', that is remarks containing information on prophylactic therapy, or relevant or closely related to it. A transcript was made of all the comments by the participants on preventive treatment. Subsequently these comments were grouped independently by three researchers. The identified themes were written out and then organised into categories and (sub-) themes by the principal investigator, according to the rules of 'thematic analysis [16-18]' into a draft analytical framework. This framework was consecutively discussed and decided upon with the other members of the team.

When there was disagreement between researchers in the analysis, the theme was analysed again by the disagreeing researchers and in case of a persisting discrepancy consensus was soughed and reached between the researchers. The analysis was coordinated by the principal investigator, who did put the remaining questions each time to both the other researchers. An interpretative analysis of the data with the help of this framework enabled the identification of several related but separate topics of experience and reasoning regarding prophylactic treatment for migraine and a tentative model for understanding patients' decision making regarding such treatment.

## Results

Five main categories of themes emerged from the focus group meetings.

### 1) Previous steps taken to prevent migraine

With regard to preventive measures, many participants were concerned that migraine was not well understood, and some found it hard to rely on prophylactic therapy because the mechanism was still unclear to them.

Almost all patients had experimented with behavioural, lifestyle or dietary actions, mostly without success and later therefore abandoned. However, some patients continued with these behaviours, even when they believed that they probably provided no benefit. Many participants avoided certain foods and other types of products. Some used specific products in order to promote their health.

'Stabilizing the biological clock', i.e., developing a stable day-night rhythm, was a widely used precaution by more than half of participants. Interventions were often supported by their physicians. For some patients, prophylaxis was the last resort.

*"I 'did' the whole alternative circuit. I tried everything. Only after all that was I ready for prophylaxis." *(Group 1, PT 1)

Many types of complementary medicines had been or were being used. Most patients believed that although prophylactic treatment is only moderately effective, it is still more effective than complementary therapies. Using a complementary therapy often hampered patients from considering prophylaxis; they were waiting for the effects of the complementary interventions. Once complementary therapies had failed, they were more willing to try regular therapies.

*"In the beginning, when my migraines were first diagnosed, we tried everything and every therapy to treat the attacks. Later on, I stopped making appointments for my migraines, I was so disappointed ... and I tried everything myself, avoided all kinds of food, gulped down vitamins and other supplements, relaxation therapies*, etc., etc." (Group 3, PT 5)

### 2) Satisfaction with current migraine treatment

Migraine patients differed in how they determined whether or not they were satisfied with their treatment. Some patients were satisfied when they were able to keep on functioning at work or at home, others were only satisfied when the headache disappeared.

A few participants kept a highly structured diary to ascertain whether there were any factors that influenced their migraine. Keeping a diary made patients more accessible to prophylactic medication.

*"My GP gave a kind of brochure; later on I continued keeping record of my headaches. I think that's very important; noting parallel things, food and so, looking back whether medication works." *(Group 1, PT 1)

According to the patients, preventing the overuse of attack treatment was only occasionally considered by the GP. According to the patients, almost no GP used that argument in the discussion about whether or not to start preventive treatment. Remarkably, some patients who used excessive attack treatment mistakenly called it 'prevention'. In their incorrect but exemplary way of thinking, they considered it to be prophylaxis because they used the attack treatment before a migraine attack occurred. Some patients showed very limited awareness about the risks of overuse of attack treatment.

*"I already take so many medications, so don't do that preventive thing to me. When I feel a headache coming, I just take a tablet and that's prevention to me." *(Group 2, PT 3)

Most patients agreed that effective migraine treatment consists of effective attack management in addition to effective prophylaxis. More than half of the patients wanted to reduce the use of attack treatment, because they felt they were using too many triptans or painkillers. However, patients still focused on the importance of attack treatment; prophylaxis took second place. This focus on attack treatment hampered their thinking about other strategies to reduce the burden of migraine.

*"I'm afraid of the side-effects of triptans; that's making me more open to prophylaxis." *(Group 1, PT 5)

The feeling of being in control of the migraine and not being controlled by it was considered a very important factor. Participants accepted a high frequency of migraine and/or long-lasting attacks as arguments for prophylaxis. However, the vast majority believed that if the attack treatment was extremely effective, there would be no need for prophylaxis. This was irrespective of the number of attacks and was in relation to what patients found 'normal' for them.

*"I'm not stuffing my body with medication when I have an attack 3 times a month, even if it is terribly intense, but when it's good treatable." *(Group 2, PT 7)

### 3) Taking the initiative for prophylaxis

Although not every patient had personal experience with migraine prophylaxis, almost everyone knew about its existence. Most patients received information from family members, physicians, the Internet, the media or pharmacists. Many participants had searched the Internet for specific information on prophylaxis and encountered both positive and negative information such as stories of patients who have had a lot to benefit from prophylaxis and others who had no good effect and suffered from significant side effects.

*"I'm using preventive therapy now. I didn't hear anything about it from the doctor ... I found out myself that something like that was available. It was in a women's magazine, not via the GP. I'm unhappy about that..."*(Group 3, PT 7)

Testimonies of other patients or information of a patient headache association was not clear enough or too ambiguous to make a first step. It did not have a direct influence on their own health-seeking behaviour.

*"Anti-epileptics, that sounds dreadful. The sort of thing you associate with lying on the ground with foam around your mouth." *(Group 2, PT 3)

There was no consensus as to who should take the initiative for prophylaxis. About half of the patients expected an active approach from their GP. Others (more urban and/or more highly educated) preferred to take the initiative themselves. All patients expected that their GP should be able to discuss the advantages and disadvantages of prophylaxis.

Patients found it important that discussion about prophylaxis should take place at the appropriate moment. This was not necessarily at the initial diagnosis, but when the patient knew more about the impact of migraine and the effectiveness of attack treatment. The need for prophylaxis could then be considered within a more realistic context.

*"I never wanted it; I'm not a pill swallower. But I find it terrible to have to call my colleagues that I have another attack again. Then they stare at me with negatively loaden, piercing eyes. And I have started to think differently about daily treatment." *(Group 2, PT 6)

Prophylaxis was often discussed when patients indicated they were no longer able to cope with the headache attacks.

*"My migraines were so severe that I went to the doctor ... I couldn't do anything but cry. He tried to comfort me and offered prophylaxis." *(Group 3, PT 4)

For a few participants, the initiative for prophylaxis was taken by the GP based on the amount of prescribed attack medication; these GPs actively monitored the use of triptans and painkillers. When confronted with such an active approach, the patients were initially cautious but subsequently regarded the GP's intervention as positive. Ultimately, almost all patients desired to have their own control over the final decision.

*"Sometimes I'm afraid he'll phone again ... because I take too much medication. I once phoned for a repeat prescription, but the doctor called back and said: You've used too much this month. Then he mentioned preventive therapy. It feels OK, that he's concerned about me." *(Group 1, PT 5)

### 4) Assessing the advantages and disadvantages of prophylaxis

From the patient's perspective, the decision to start prophylaxis is complex. There is a wide range of perceived advantages and disadvantages, migraine patterns often vary, and the underlying concerns also differ.

*"The pattern of attacks of my migraine is too weird to be able to figure out whether prophylaxis will help me or not." *(Group 3, PT 2)

*"Accepting prophylaxis is difficult, because my attacks sometimes stay away for a long time. It's sometimes months before I have another attack, but once they start they're very frequent." *(Group 3, PT 3)

*"I'm now using so many triptans ... this can't be a good thing." *(Group 3, PT 2)

*"I just don't want to do it. I'm very anti-drugs." *(Group 3, PT 1)

When considering prophylaxis, all patients experienced negative or obstructive elements, as well as positive factors. Participants had differing views on this subject, some mainly emphasised the positive aspects and others mainly the negative aspects.

The most important negative factors were the fear of side-effects, the assumption that prophylaxis will have little impact, and the feeling of becoming a chronic patient. The issue of 'becoming a chronic patient' was expressed in all sessions, and about 50% of the patients associated the use of prophylactic drugs with 'old age' and 'chronic disease'. Participants emphasised that they did not feel like a 'patient' in between the migraine attacks, so it did not feel appropriate to use medication on a daily basis. Despite a high impact of migraine and although many (daily) preventive measures and behavioural adaptations has been adopted, the use of prophylactic drugs was not easily accepted.

More than half of the patients stated that daily use of tablets for migraine would make them feel emotionally unhealthier. Other negative factors included the fear of drug dependency, a low assessment of their own capacity for compliance, and the negative reactions of persons in their direct surroundings.

*"If I were to take tablets every day, I'd feel like I'm a patient. Now I just have a headache sometimes ... actually it's many times." *(Group 3, PT 3)

*"I think I'd forget it (medication) so often that it wouldn't be effective." *(Group 3, PT 7)

*"I'm afraid of becoming dependent on those drugs." *(Group 3, PT 2)

*"It's something in the head about not wanting to take tablets every day." *(Group 2, PT 4)

*"When you receive preventive therapy for something, people think you're a pitiful case." *(Group 2, PT 6)

*"The question is: how does migraine affect your life. I don't want migraine to affect my life, and taking drugs every day would have a major effect on my life." *(Group 1, PT 6)

The factors that contribute to positive decision appear to rest on a more calculated way of thinking or approach; weighing the advantages against the disadvantages and assessment of the degree of effectiveness.

Half of the participants had benefited from prophylaxis. The main positive benefits were a reduction in the burden of migraine with an increase in the range of abilities; this was particularly important when the impact was high. Other positive features were the ease of administration, an overall general gain in health, a reduction in acute medication, less confrontations with the GP in case acute medication was used excessively, and less pressure from others close to them. When the benefits were clearer, patients were able to accept prophylaxis or were at least willing to try it. Most of the patients stated they would accept daily drug intake if their migraine frequency would be halved.

*"I don't care what I have to do; I'd do anything to get rid of my headaches." *(GR 2, PT 4)

*"If it worked for 100%, I would certainly join the users!" *(Group 3, PT 3)

*"With prophylactic drugs you're able to participate much more in sport activities - which I enjoy very much." *(Group 1, PT 4)

*"If somebody said to me: "The migraines will disappear if I cut off your hand", then I'd say: Cut off my whole arm!" *(Group 3, PT 3)

Many patients anticipated reimbursement problems with the healthcare insurance companies when receiving prophylactic therapy (in fact, in the Netherlands, all costs of prophylactic therapies are fully covered by healthcare insurance for all patients). Patients who had experience with prophylaxis reported that they had no problems with health insurance or the financial side of treatment costs.

Apart from the duration of the attack another important factor was the situation involved, e.g. being at school, at work, or with friends or family. For similar attack rates the perceived need for prophylaxis differed between patients.

Not being able to take care of others was a strong positive factor for prophylaxis. Apart from the impact of migraine on themselves also the impact on other persons for whom they are responsible (e.g. children, family members, colleagues, etc.) was an important argument for preventive treatment.

*"I can't even make it to the meetings of my sports club. I might manage it once, but the second, third and fourth time they wouldn't understand. When you feel that negative impact from migraine, then you really want to start thinking about preventive treatment." *(Group 3, PT 4)

### 5) The relationship with the physician and the feeling to be heard

At the time of diagnosis, being taken seriously about the burden of the migraine and acknowledgement of their suffering was considered most important. But this was not the appropriate time when patients were interested in prevention. Many migraine patients felt there was a limit to the extent to which their physician is able to comprehend the burden they bear. They considered that their GP unable to imagine how difficult it is to experience a migraine attack, whereas others mentioned a sympathetic response from their GP. Patients indicated that at a later stage a good empathetic relationship with the doctor was important for the acceptance of prevention.

*"He (GP) was really concerned about me, about the enormous number of attacks I had. That was good and very considerate of him." *(Group 1, PT 4)

*"I think that he (GP) thinks: what on earth can I do for you anyway..." *(Group 2, PT 2)

*"I found that now something really has to be done ... so I went to the doctor. He said: It sounds like classic migraine; we'll see what we can do. I should have done this much earlier ... at last I felt that someone understood." *(Group 1, PT 2)

*"There's always that fear of the next attack, and my family doctor seemed to understand that fear. First and foremost, you have to be taken seriously by your doctor." *(Group 1, PT 1)

An important influence was the way their GPs treated them. Positive factors in promoting prevention were having a positive interaction and the feeling being taken seriously. On the other hand, being dissatisfied about the approach of the physician hampered the willingness to consider prophylaxis.

*"Primarily I want to be taken seriously, but I can not complain. He's handled it well, with the start of preventive treatment." *(Goup1 PT 5)

*"If you have more than two attacks a month, they just give you a prescription for anti-epileptics and - before you know it - you're outside again." *(Group 3, PT 6)

## Discussion

### Summary of main findings

The present study describes patients' subjective opinions about prophylaxis as a treatment option for migraine.

**Figure 1:**
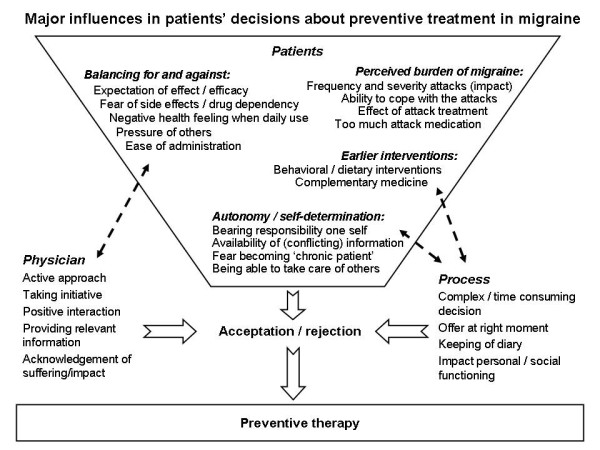
**Major influences in patients' decisions about preventive treatment in migraine**.

A number of conditions that must be met before preventive therapy is accepted and that these often are related to each other (Figure 1). These conditions can be patient related, clinician related or be related to the disease or the disease process. Knowledge on the importance of these issues for the decision making of patients is crucial for physicians dealing with migraine patients in daily practice.

Patients indicate a number of important factors in favour of the use of prophylaxis related to the perceived burden of migraine; a high frequency of attacks, severe attacks, and lack of effectiveness of attack treatment. These are characteristics of the migraine itself, on which physicians do not have much influence (however they have certainly on proper attack treatment). The patient makes a balance of pros and cons. Expectations of the beneficial effects, fear of side effects and drug dependency and negative health feeling in case of daily use of medication, play a considerable role in making this balance.

The willingness to try prophylaxis increased after other interventions had been tried (e.g. dietary changes, changes in lifestyle or previous complementary treatments). Patients prefer strongly to take the decision themselves and want to have responsibility themselves. The individual history of earlier interventions is pivotal. Several factors increase the resistance to accepting prophylaxis, such as changing the scope from seeing migraine as an intermittent to seeing it as a chronic disease.

When weighing the facts and reaching a decision on prophylaxis, the physician has a major influence, especially by providing relevant information. In the process of getting more insight on their migraine, patients feel that the physician can be helpful. Patients attach great importance to a good and trusted relationship with the physician and often prefer an active approach.

It is important to acknowledge that a patient is going through a process, and in time tend more and more towards a decision. It takes time to realize that one has a severe problem with migraine and that the migraine has a large impact. Keeping a diary can provide such an insight in an earlier stage [19]. From the management perspective, patients need to be receptive to the idea of prophylaxis at the right moment in their migraine history.

### Study strengths and limitations

This focus group research aimed to explore opinions through a purposeful sample covering a range of subjects and doesn't provide numbers and clear conclusions such as quantitative research.

We decided to have separate male and female groups based on the assumption that their approaches to migraine differ, and that a mixed group may inhibit the exploration of some key elements of migraine for woman, such as a relation with menstruation.

Saturation of themes occurred within the three groups, when no new themes arose that had not been included in our topic guide. Within the groups, a diversity of approaches was found. For example, the urban group was more highly educated and displayed more resistance to prophylaxis, and needed a more rational and evidence-based requirement for its introduction.

The rural group had a more passive attitude and indicated more acceptance to the propositions from their physician.

A weakness of the present study is that it was conducted in the Dutch language and is reported in English. Qualitative studies aim to capture meaning from the narratives of respondents and some loss and/or distortion may have occurred in the translation process. However, we had the Dutch texts translated by two experienced translators and from the perspective of migraine a native English speaking expert physician on migraine looked into the patient remarks.

### Comparison with other studies

Parallel to this study another qualitative study from the same research group, also on prophylaxis for migraine, explored the attitude of GPs [20]. That study confirmed the complexity of the decision-making process, which from the perspective of the GP was also not based on the impact of migraine alone. Patients and GPs showed a similar degree of hesitance, not because of lack of knowledge or lack of interest, but because of doubts about effectiveness, side-effects, and the risk of developing drug dependence.

Of the qualitative studies on migraine, only one has addressed preventive therapy [21]. In that study by Rozen, the method (questionnaire) and setting (third-line centre) were different to ours and all patients had prior exposure to migraine prophylaxis. The decision whether or not to start preventive therapy had already been made, and the questionnaire mainly addressed side-effects and the choice of drugs. That study provided no information on its aims or how the decision concerning prophylaxis was made.

Two qualitative studies show agreement with our study in relation to prevention. One study shows remarkable similarities on the patient communication with the GP and on the search for complementary therapies by patients. Because this study is not about prevention, is does cover the influence of these two issues on prevention [22]. The other study reports that self-efficacy scores were positively associated with the use of positive psychological coping strategies to prevent headaches [23].

Other studies which address patient factors in migraine do not address prophylaxis and focus on the needs of migraine patients [24,25], decision-making in migraine [26-28], the burden of migraine and quality of life [29,30], perimenopausal headache [31], migraine in midlife women [32], and pressure on patients related to referral [33], and therefore have almost no overlap to our study. The questionnaire study by Kowacs et al. on the patients' view on side effects of preventive treatment revealed that side effects are better accepted by patients with high use or actual overuse of attack treatment, which is consistent with our findings [34]. The questionnaire study of Kol et al. found that 55% of patients with two or more attacks per month wanted to use prophylaxis, while only 8% actually used this treatment. This paradox is one of the underlying themes in our study [12].

Similar studies have also been conducted with other chronic diseases. For example, the study of Adams et al. on acceptance of the preventive treatment for asthma [35]. Specific for prophylactic asthma treatment is that it is given even in asymptomatic periods, the inhalation therapy is visible to others and there is fear for side effects on the longer term ('steroid fear'). In contrast, in migraine only patients with frequent and severe attacks are treated, mainly the side effects on the short term are feared, and in general patients have no trouble with the acceptance of the migraine as such. This comparison shows that migraine has similarities, but also differences with other chronic diseases. Most likely the opinions of patients differ per indication.

### Conclusions

The benefits of prophylactic medication for migraine are under-exploited. Future research should focus on the various aspects involved in decisions about preventive treatment, as reflected in the present study. An understanding by physicians of the patient's feelings and concerns towards prevention are important for more effective use of these agents. When advising migraine patients on prophylaxis it is important to explicitly address their underlying thoughts and emotions, and to consider the intervention at an appropriate moment in the course of the patient's migraine experience.

## Competing interests

Dr. M.D. Ferrari has received unrestricted research grants from, or served as a consultant to Almirall Prodesfarma, AstraZeneca, GlaxoSmithKline, Merck, Pfizer, Menarini, Johnson and Johnson, and Pharmacia. Dr. D. Kernick has acted in an advisory capacity to MSD and AstraZeneca. Dr. F. Dekker has received an unrestricted research grant from Janssen-Cilag, with no relation to the present study. All other authors state that there are no competing interests.

## Authors' contributions

Original idea and conception of the study: FD, AKN. Development of the protocol: FD, AKN, WJJA. Organization and participation in the focus groups: FD, AKN. Qualitative analysis: FD, BA, AKN, RR. DK and RR participated in the study design and helped to draft the manuscript. Writing of the manuscript: FD. All the authors have read the draft critically, have made contributions, and have approved the final text.

## Pre-publication history

The pre-publication history for this paper can be accessed here:

http://www.biomedcentral.com/1471-2296/13/13/prepub
